# Kalman-Filter-Based Learning of Characteristic Profiles of Lithium-Ion Batteries †

**DOI:** 10.3390/s22145156

**Published:** 2022-07-09

**Authors:** Masahito Arima, Lei Lin, Masahiro Fukui

**Affiliations:** 1 Research & Development Strategy Department, Daiwa Can Company, Sagamihara 252-5183, Japan; l-lin@mail.daiwa-can.co.jp; 2 Graduate School of Science and Engineering, Ritsumeikan University, Kusatsu 525-8577, Japan; mfukui@se.ritsumei.ac.jp

**Keywords:** lithium-ion battery, efficiency degradation, reusable battery, degradation diagnosis, battery aggregation, Kalman filter

## Abstract

The main analyzed aspect of lithium-ion battery (LIB) degradation so far has been capacity fading. On the other hand, interest in efficiency degradation has also increased in recent years. Battery aggregation, which is expected to absorb the surplus of variable renewable energies such as photovoltaic energy, is affected by efficiency degradation in terms of the decreases in the economic gain and renewable energy use. Reusable LIBs could be used as aggregation components in the future; naturally, the variety of charge–discharge efficiencies might be more complex. To improve the operation efficiency of aggregation, including that obtained using reusable LIBs, we propose the Kalman-filter-based quasi-unsupervised learning of the characteristic profiles of LIBs. This method shows good accuracy in the estimation of charge–discharge energy. It should be emphasized that there are no reports of charge–discharge energy estimation using the Kalman filter. In addition, this study shows that the incorrect open-circuit voltage function for the state of charge, which is assumed in the case of a reused battery, could be applied as the reference for the Kalman filter for LIB state estimation. In summary, it is expected that this diagnosis method could contribute to the economic and renewable energy usage improvement of battery aggregation.

## 1. Introduction

Climate change is considered a global problem. Thus, the use of variable renewable energies, such as photovoltaic and wind energies, has increased [1]. Consequently, problems associated with surplus electric energy have emerged. A widely known problem is the distinctive electric demand chart called “the duck curve”, which depicts daytime photovoltaic overgeneration [2]. This is the substantial electric demand profile with a characteristic duck-like shape. That is, the bulge in the abdominal region of the demand profile represents a mass generation of photovoltaic energy, indicating an overgeneration risk by transcending the abilities of thermal power generation control and demand generation. In fact, the large-scale reductive control of variable energies has been implemented, for example, in Kyushu (since October 2019), Shikoku and Tohoku (since April 2022), and Hokkaido (since May 2022) in Japan. Naturally, the results include the loss of economic and environmental benefits despite capital investments in renewable energy.

Among the expected solutions are aggregated rechargeable batteries [3] supported by the Internet of Things. That is, surplus renewable energy could be charged and then discharged at a later time of increased demand. The lithium-ion battery (LIB) is an important candidate that has been adopted as a component of electric vehicles and stable energy storage systems. To reduce resource demand, lower prices, and promote a more commercial (and widespread) use of LIBs, many considerations of reuse [4] have been carried out. Consequently, a new international standard, IEC 63330, for the requirements for the reuse of LIBs is being discussed, with expected issuance at the end of 2023 [5]. The implication is that many aggregated rechargeable batteries consisting of reused LIBs could be popular in the future.

Degradation is an important characteristic of LIBs, as performance declines depending on charge–discharge cycle times and storing periods [6,7,8]. A decrease in the full-charge capacity (FCC) and its directly related indices, such as the state of health (SOH) and the remaining useful life (RUL), have been discussed as the main aspects of the degradation of LIBs. Therefore, the capacity-degradation diagnosis and RUL prediction of LIBs have been extensively studied. Equivalent circuit models and AC impedance analysis can measure the change in internal impedance details, which can be treated as an indicator of the FCC [9,10]. The charge–discharge voltage difference indicates the RUL [11,12]. Differential voltage [13,14] and charge curve [15] analyses clarify the internal state of LIBs (e.g., losses of lithium inventory and active materials). Adaptive filters such as the Kalman filter [16] and recursive least square [17] can accurately estimate the state of charge. Thus, the FCC can be calculated. Neural networks can estimate the SOH or RUL from chronological data [18,19]. On the other hand, a decrease in the charge–discharge energy efficiency, called “efficiency degradation”, has been recently reported as another aspect of degradation [20]. It causes the increase in energy loss during LIB operation, and it is an important factor for large-scale LIBs [21]. It induces the order effect of charging in the case of aggregated multiple LIBs [22]. This can be understood in the following way: The degradation degrees of LIBs differ because of the conditions of operation. In addition, aggregated batteries could include various LIB models, manufacturers, and reused histories, indicating that each LIB has various characteristics of charge–discharge energy efficiency. The operation of a high-efficiency LIB could result in a smaller loss of energy and greater economic gain. How to find a highly efficient LIB is important for the economic and energy-saving operation of battery aggregation. Furthermore, an efficiency-based scrapping criterion of LIBs has been proposed [23]. That is, an efficiency-degradation diagnosis is necessary instead of a capacity diagnosis. In addition, it is necessary that this diagnosis be carried out during battery aggregation. It is also desirable that this diagnosis is online and unsupervised (without a pre-cycle test) because of the variety of the characteristics of aggregated LIBs.

Cycle energy efficiency is defined as the ratio of discharge energy to charge. Therefore, an estimation of these values is required for an efficiency-degradation diagnosis. The formula for charge–discharge energy consists of the FCC, open-circuit voltage (OCV), internal resistance (R), and state of charge (SOC) [24]. Especially, the functions of the OCV and R profiles of the SOC are required. There are many reported methods for estimating the OCV profile, and they can be divided roughly into two groups. In the first group—pseudo OCV (pOCV) [25]—the measuring process involves the cycling of an LIB once with more than 10 h at a constant current (<0.1 C) in the range from full discharge to full charge. Then, the OCV profile is calculated as the mean values of the charge–discharge voltages. The second group is the galvanostatic intermittent titration technique (GITT) [26], with a square wave, charge–discharge current, and long-time relaxation between each wave. The estimated R profile can be calculated by dividing the differences between the charge–discharge voltages and OCVs by the current. That is, the R profile can be estimated with the pOCV or GITT measuring process. However, the conditions of the pOCV and GITT include a very long current control span and are quite different from battery aggregation. Therefore, these cannot be adapted for OCV profile estimation during operation.

The Kalman filter, based on an electrochemical model, is widely known as an online estimation method of LIB state parameters—mainly the SOC, polarization voltage, and ohmic resistance [27,28]. A dynamic SOC estimation (the main purpose) could be accurate and important for the profile estimation of the OCV and R. However, the OCV and R estimated by the Kalman filter are discrete scalars corresponding to a specific SOC in most cases. Further, OCV profile estimations using the Kalman filter are less frequently studied. Moreover, a high accuracy acquisition in keeping with the SOC estimation accuracy is difficult to establish [29]. Given the relevance of the OCV, the same is true for R profile estimation.

Based on the aforementioned problems, the purpose of this study was the establishment of a new method for estimating the characteristic profiles of LIBs based on a collaboration between the extended Kalman filter and the process of graph deformation by Gaussian function addition. For this purpose, charge–discharge energy estimation and efficiency-degradation diagnosis were carried out using the SOC, OCV, and R estimated by the extended Kalman filter. The effectiveness of this method based on initial data assumed as the various characteristics of aggregated batteries was verified.

## 2. Materials and Methods

### 2.1. Test Sample and Details of Charge–Discharge Cycle Test

In this study, an 8-series LIB module was cycled, and the charge–discharge data were acquired in chronological order. The details of the cycle test are described in Table 1. During the cycle test, constant power mode was adopted for simulating the condition of charge–discharge performed by a power conditioning system. In research studies of degradation diagnosis and state estimation, cycle tests of small LIB cells, such as 18,650 (e.g., 3.6~3.7 V, 0.7~3.5 Ah, typical), are mainly adopted [9,10,11,12,14,16,17,19]. In contrast to this, an 8-series LIB module composed by large cells of 50 Ah was adopted as the component of an aggregated battery pack in this study. The atmosphere temperature of the cycle test was set as room temperature, although the temperature of the module fluctuated in the range from about 20 to 30 °C because of the inevitable internal resistance heat of charge and discharge. In this study, we aimed to acquire 500-cycle degradation data, which correspond to the industrially standardized cycle durability test data [30]. In addition, we considered an initial cycling capacity recovery effect of about 150–200 cycles [31]. Therefore, we adopted 700 cycles of charge–discharge.

### 2.2. Profile-Graph Deformation by Gaussian Function Addition

The profile-graph deformations of the OCV and R were carried out by height-adjusted Gaussian function addition [32]. These discretized profile data corresponding to multiple SOC values were prepared as vectors.

(1) $$D={\left(v_{1},\;v_{2},\;\cdots ,v_{101}\right)}^{⊺}$$

where D is the representative vector applied to both the OCV and R. The element number of vector D was set as 101, corresponding to an SOC of [0, 0.01, 0.02, ⋯,1]. The amount of graph deformation was defined using the a priori estimate of error $\tilde{E}$ of a target physical quantity.

(2) $$M_{l}=\tilde{E}Lexp\left(-\frac{{\left(10^{-2}\left(l-1\right)-S_{oc,0}\right)}^{2}}{2\sigma^{2}}\right),\;\;1\leq l\leq 101,\;\;l\in \mathbb{Z}\;$$

The center coordinate of deformation on the SOC axis is $S_{oc,0}$, namely, the time of $\tilde{E}$ extraction from cycle data is when the SOC is $S_{oc,0}$. L is the learning rate; therefore, it should be in the range of 0<L≤1. σ is the value representing the standard deviation, that is, the rough range of graph deformation; therefore, too large a value is not desirable. l is the order number to specify an element of D. Namely, the overall deformation amounts can be presented as shown below.

(3) $$M={\left(M_{1},\;M_{2},\;\cdots ,M_{101}\right)}^{⊺}$$

**Figure 1:**
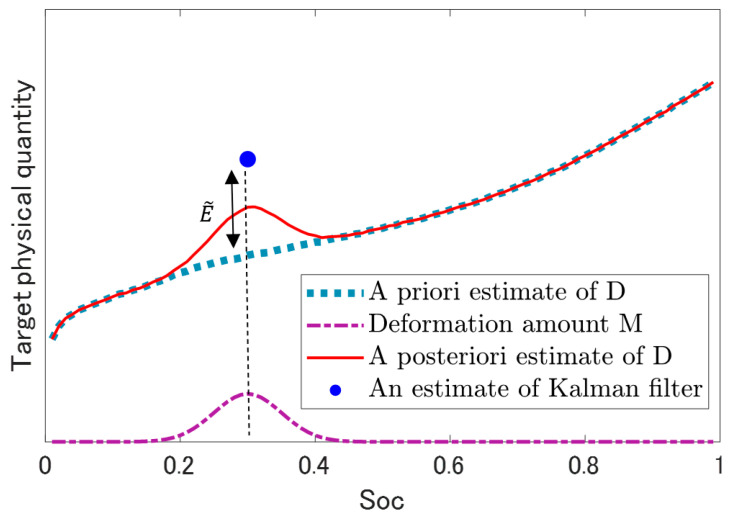
Image of profile-graph deformation.

In summary, the process of this profile-graph deformation is presented as shown below.

(4) $$\hat{D}={\hat{D}}^{-}+M$$

where ${\hat{D}}^{-}$ is the a priori estimate and $\hat{D}$ is the a posteriori estimate of D. The image of this graph deformation process is shown in Figure 1. In addition, the above was repeatedly carried out during the overall charge–discharge cycles, that is, on various SOCs. $\tilde{E}$ was calculated using the Kalman filter as shown below.

### 2.3. Algorithm of Kalman Filter

**Figure 2:**
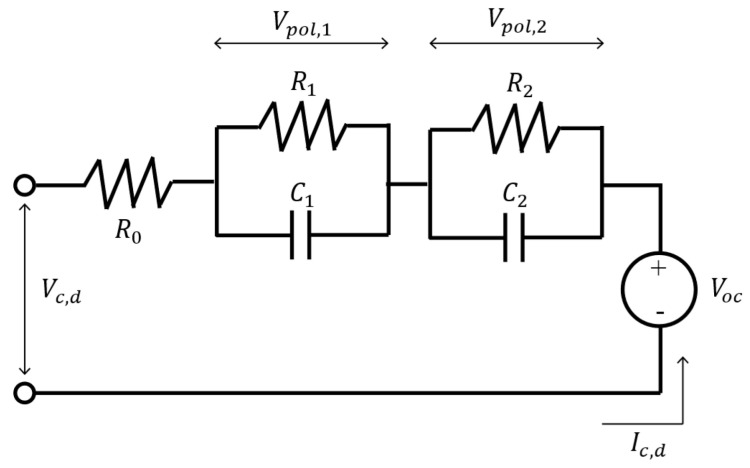
Equivalent circuit model of an LIB.

In this research study, we adopted an extended Kalman filter adjusted for the state estimation of LIBs [33]. A state equation and an observation equation were formularized as the linearized discrete-time system.

(5) $$x_{k+1}=A_{k}x_{k}+B_{k}u_{k}+w_{k}$$

(6) $$y_{k}=C_{k}x_{k}+v_{k}$$

where $x_{k}$ is a state space vector; $y_{k}$ is an observation value; $w_{k}$ is a normally distributed system noise, where the average is 0 and the variance is $\sigma_{w}{}^{2}$; and $v_{k}$ is a normally distributed observation noise, where the average is 0 and the variance is $\sigma_{v}{}^{2}$. These equations were set based on the equivalent circuit model of LIBs described in Figure 2. Therefore, Equations (7)–(9) could be formularized.

(7) $$C_{1}\frac{dV_{pol,1}}{dt}+\frac{V_{pol,1}}{R_{1}}=I_{c,d}$$

(8) $$C_{2}\frac{dV_{pol,2}}{dt}+\frac{V_{pol,2}}{R_{2}}=I_{c,d}$$

(9) $$V_{c,d}=V_{oc}+V_{pol,1}+V_{pol,2}+I_{c,d}R_{0}$$

where $R_{o}$, $R_{1}$ and $R_{2}$ are resistances; $C_{1}$ and $C_{2}$ are capacitances; $V_{pol,1}$ and $V_{pol,2}$ are polarization voltage values of each RC parallel unit; $V_{c,d}$ is the terminal voltage during charge–discharge; $I_{c,d}$ is the charge–discharge current—note that it is defined as a negative value during discharge; and $V_{oc}$ is the OCV value. Equations (7) and (8) can be discretely transformed into Equations (10) and (11) by the forward Euler method.

(10) $$V_{pol,1}\left(k+1\right)=\left(1-\frac{\Delta t}{R_{1}C_{1}}\right)V_{pol,1}\left(k\right)+\frac{I_{c,d}}{C_{1}}\Delta t$$

(11) $$V_{pol,2}\left(k+1\right)=\left(1-\frac{\Delta t}{R_{2}C_{2}}\right)V_{pol,2}\left(k\right)+\frac{I_{c,d}}{C_{2}}\Delta t$$

where Δt is the time resolution between time series k and k+1. Based on Equations (10) and (11), equations of state space (5) and observation (6) could be formularized as (12)–(13).

(12) $$x_{k+1}=\left[\begin{array}{c} S_{oc}\left(k+1\right)\\ V_{pol,1}\left(k+1\right)\\ \begin{array}{c} V_{pol,2}\left(k+1\right)\\ R_{0}\left(k+1\right) \end{array} \end{array}\right]=\left[\begin{array}{cccc} 1 & 0 & 0 & 0\\ 0 & 1-\frac{\Delta t}{R_{1}C_{1}} & 0 & 0\\ 0 & 0 & 1-\frac{\Delta t}{R_{1}C_{1}} & 0\\ 0 & 0 & 0 & 1 \end{array}\right]\cdot \left[\begin{array}{c} S_{oc}\left(k\right)\\ V_{pol,1}\left(k\right)\\ \begin{array}{c} V_{pol,2}\left(k\right)\\ R_{0}\left(k\right) \end{array} \end{array}\right]+\left[\begin{array}{c} \frac{\Delta t}{C_{fc}}\\ \frac{1}{C_{1}}\\ \frac{1}{C_{2}}\\ 0 \end{array}\right]\cdot I_{c,d}\left(k\right)+w_{k}$$

(13) $$y_{k}=V_{c,d}\left(k\right)=V_{oc}\left(k\right)+V_{pol,1}\left(k\right)+V_{pol,2}\left(k\right)+I_{c,d}\left(k\right)R_{0}\left(k\right)+v_{k}$$

Equation (12) is composed of linear functions. On the other hand, Equation (13) is a nonlinear function because of $V_{oc}\left(k\right)$, which is a function of the SOC, that is, a parameter that should be estimated by the pOCV [25] or GITT [26] in the conventional case. For the adoption of a nonlinear observation equation in the Kalman filter, Equation (13) was differentiated as shown below.

(14) $$y_{k}=V_{oc}\left(k\right)+{\left[\begin{array}{c} {\frac{dV_{oc}}{dS_{oc}}|}_{S_{oc}={\hat{S_{oc}}}^{-}\left(k\right)}\\ 1\\ \begin{array}{c} 1\\ I_{c,d}\left(k\right) \end{array} \end{array}\right]}^{⊺}\;\left[\begin{array}{c} S_{oc}\left(k\right)\\ V_{pol,1}\left(k\right)\\ \begin{array}{c} V_{pol,2}\left(k\right)\\ R_{0}\left(k\right) \end{array} \end{array}\right]+v_{k}$$

That is to say, $A_{k}$, $B_{k}$, and $C_{k}$ in Equations (5) and (6) were set as shown below.

(15) $$A_{k}=\left[\begin{array}{cccc} 1 & 0 & 0 & 0\\ 0 & 1-\frac{\Delta t}{R_{1}C_{1}} & 0 & 0\\ 0 & 0 & 1-\frac{\Delta t}{R_{1}C_{1}} & 0\\ 0 & 0 & 0 & 1 \end{array}\right],\;B_{k}=\left[\begin{array}{c} \frac{\Delta t}{C_{fc}}\\ \begin{array}{c} \frac{1}{C_{1}}\\ \frac{1}{C_{2}}\\ 0 \end{array} \end{array}\right],\;C_{k}={\left[\begin{array}{c} {\frac{dV_{oc}}{dS_{oc}}|}_{S_{oc}={\hat{S_{oc}}}^{-}\left(k\right)}\\ 1\\ \begin{array}{c} 1\\ I_{c,d}\left(k\right) \end{array} \end{array}\right]}^{⊺}$$

where $R_{1}=R_{2}=10^{-4}$, $C_{1}=10^{4}$ and $C_{2}=10^{5}$ were set as the initial conditions. Based on the charge–discharge cycle conditions, Δt was set as 10. The differentiation of $V_{oc}$ by $S_{oc}$ was calculated based on the regressed polynomial of the OCV profile described in the following subsection.

Two important steps of the Kalman filter were carried out in the following way: At first, in the estimation step, the a priori estimates of state space vector ${\hat{x}}^{-}{}_{k+1}$ and error covariance matrix $P_{k+1}{}^{-}$ were calculated by Equations (16) and (17).

(16) $${\hat{x}}^{-}{}_{k+1}=A_{k}{\hat{x}}_{k}+B_{k}I_{c,d}\left(k\right)$$

(17) $$P_{k+1}{}^{-}=A_{k}P_{k}{}^{-}A_{k}{}^{⊺}+\sigma_{w}{}^{2}$$

Following this, in the filtering step, Kalman gain $g_{k+1}$, a posteriori estimate state space vector ${\hat{x}}_{k+1}$, and error covariance matrix $P_{k+1}$ were calculated by Equations (18)–(20).

(18) $$g_{k+1}=\frac{P_{k+1}{}^{-}C_{k+1}{}^{⊺}}{C_{k+1}P_{k+1}{}^{-}C_{k+1}{}^{⊺}+\sigma_{v}{}^{2}}$$

(19) $${\hat{x}}_{k+1}={\hat{x}}^{-}{}_{k+1}+g_{k+1}\left(y_{k+1}-{\hat{y}}^{-}{}_{k+1}\right)$$

(20) $$P_{k+1}=\left(I-g_{k+1}C_{k+1}\right)P_{k+1}{}^{-}$$

Initially, ${\hat{x}}_{0}=0$ and $P_{0}=0$ were given as the tentative vector and matrix. Then, the steps of Equations (16)–(20) were continuously carried out by substituting the elements of $A_{k}$, $B_{k}$, and $C_{k}$ that were taken from the values of the cycle chronological data and parameters of the previous step. From these steps, the values of the SOC, OCV, and R were continuously estimated; simultaneously, the estimated profile graphs of the OCV and R were deformed by Gaussian function addition as described above.

### 2.4. A Posteriori Estimates of OCV and R by Kalman Filter and Extraction of a Priori Estimate of Error for Profile-Graph Deformation

From the Kalman filter process described above, the a posteriori estimates of the OCV and R were calculated using Equations (21) and (22). R was assumed as the DC resistance and defined as the quotient of the overall polarization voltage ($V_{pol,1}\left(k\right)+V_{pol,2}\left(k\right)+I_{c,d}\left(k\right)R_{0}\left(k\right)$) and charge–discharge current.

(21) $${\hat{V}}_{oc}\left(k+1\right)=y_{k+1}-C_{k+1}{\hat{x}}_{k+1}$$

(22) $$\hat{R}\left(k+1\right)=\frac{y_{k+1}-{\hat{V}}_{oc}\left(k+1\right)}{I_{c,d}\left(k+1\right)}$$

The a priori estimates of errors ${\tilde{V}}_{oc}\left(k\right)$ and $\tilde{R}\left(k\right)$, which correspond to $\tilde{E}$ in Equation (2), were calculated by the differences between the a priori estimate (${\hat{V}}_{oc}{}^{-}\left(k\right)$ and ${\hat{R}}^{-}\left(k\right)$) and the estimate of the Kalman filter (${\hat{V}}_{oc}\left(k\right)$ and $\hat{R}\left(k\right)$). Approximation with a sixth-degree polynomial could be applied to both the OCV and R [34], and the discretized data of both profiles that corresponded to D in Equation (1) were regressed as polynomials to $S_{oc}$. Accordingly, ${\hat{V}}_{oc}{}^{-}\left(k+1\right)$ and ${\hat{R}}^{-}\left(k+1\right)$ were calculated by the polynomials and ${\hat{S}}_{oc}\left(k+1\right)$, which was included in ${\hat{x}}_{k+1}$, as described below.

(23) $${\hat{V}}_{oc}{}^{-}\left(k+1\right)=\overset{6}{\underset{i=0}{\sum }}K_{ocv,i}{\hat{S}}_{oc}{}^{i}\left(k+1\right)$$

(24) $${\hat{R}}^{-}\left(k+1\right)=\overset{6}{\underset{i=0}{\sum }}K_{R,i}{\hat{S}}_{oc}{}^{i}\left(k+1\right)$$

$K_{ocv,i}$ and $K_{R,i}$ are the regressed coefficients of polynomials. A priori estimation errors ${\tilde{V}}_{oc}\left(k+1\right)={\hat{V}}_{oc}\left(k+1\right)-{\hat{V}}_{oc}{}^{-}\left(k+1\right)$ and $\tilde{R}\left(k+1\right)=\hat{R}\left(k+1\right)-{\hat{R}}^{-}\left(k+1\right)$ were calculated and substituted in Equation (2) as $\tilde{E}$; simultaneously, ${\hat{S}}_{oc}\left(k+1\right)\;$ was substituted to $S_{oc,0}$.

### 2.5. Charge–Discharge Energy Estimation

The discretized data of the OCV and R that corresponded to D were continuously updated by the estimated parameters of the Kalman filter and the processes of profile-graph deformation. Coefficients $K_{ocv,i}$ and $K_{R,i}$ of polynomials (23)–(24) were regressed after each charge–discharge cycle. That is to say, polynomials ${\hat{V}}_{oc}\left(S_{oc}\right)$ and $\hat{R}\left(S_{oc}\right)$ were regressed cycle by cycle. The estimated charge–discharge energy for a cycle ${\hat{W}}_{c,d}$ could be calculated as shown below.

(25) $${\hat{W}}_{c,d}=C_{fc}\overset{1}{\underset{0}{\int }}\left\{{\hat{V}}_{oc}\left(S_{oc}\right)+{\hat{I}}_{c,d}\left(S_{oc}\right)\;\hat{R}\left(S_{oc}\right)\right\}dS_{oc}$$

where $C_{fc}$ is the FCC value of the corresponding cycle. It should be noted that $C_{fc}$ was set as the actual measured value since there are so many reports of FCC degradation diagnosis, as mentioned in the introduction of this paper. Constant power mode was applied as cycle condition, that is, the relationship between power P, ${\hat{I}}_{c,d}\left(S_{oc}\right)$, ${\hat{V}}_{oc}\left(S_{oc}\right)$, and $\hat{R}\left(S_{oc}\right)$ could be formularized as shown below.

(26) $$P={\hat{I}}_{c,d}\left(S_{oc}\right)\;\left\{{\hat{V}}_{oc}\left(S_{oc}\right)+{\hat{I}}_{c,d}\left(S_{oc}\right)\;\hat{R}\left(S_{oc}\right)\right\}$$

We should note that P is defined as a negative value during discharge in the same way as $I_{c,d}$. Hence, ${\hat{I}}_{c,d}\left(S_{oc}\right)$ could be calculated by solving quadratic Equation (26) as shown below.

(27) $${\hat{I}}_{c,d}\left(S_{oc}\right)=\frac{-{\hat{V}}_{oc}\left(S_{oc}\right)+\sqrt{{\hat{V}}_{oc}{}^{2}\left(S_{oc}\right)+4P\hat{R}\left(S_{oc}\right)}}{2\hat{R}\left(S_{oc}\right)}$$

From this, we could formularize Equation (26) as the function composed of pre-known P, ${\hat{V}}_{oc}\left(S_{oc}\right)$, and $\hat{R}\left(S_{oc}\right)$, so that ${\hat{W}}_{c,d}$ could be calculated.

In addition, ${\hat{V}}_{oc}\left(S_{oc}\right)$ was used as a reference for $C_{k}$ in the Kalman filter, so an initial ${\hat{V}}_{oc}\left(S_{oc}\right)$ had to be set artificially. The ${\hat{V}}_{oc}\left(S_{oc}\right)$ profiles of commercial LIBs could be adopted and are typically roughly classified into three types having the Co/Ni/Mn cathode, FePO_4_ cathode, and Ti anode. Although there could be subtle differences in the profiles depending on the active material composition chosen by the LIB manufacturer, the roughly set initial ${\hat{V}}_{oc}\left(S_{oc}\right)$ value could be converged by continuous learning of the Kalman filter and profile-graph deformation. This is the meaning of quasi-unsupervised learning. On the other hand, the initial value of $\hat{R}\left(S_{oc}\right)$ was set as 0, since ${\hat{x}}_{0}=0$ in the Kalman filter. $\hat{R}\left(S_{oc}\right)$ could be converged in the same way as ${\hat{V}}_{oc}\left(S_{oc}\right)$.

### 2.6. Estimation Error and Accuracy Analyses of Charge–Discharge Energy

In this study, learning rate L, graph deformation range σ, and the initial difference between ${\hat{V}}_{oc}\left(S_{oc}\right)$ and the actual OCV profile were mainly analyzed. To quantitatively analyze and compare, the actual value of the *i* th charge–discharge cycle $W_{c,d,i}$ was measured, and the corresponding estimate error, ${\tilde{W}}_{c,d,i}$,was calculated from estimate ${\hat{W}}_{c,d,i}$.

(28) $${\tilde{W}}_{c,d,i}=W_{c,d,i}-{\hat{W}}_{c,d,i}$$

Then, error index $I_{maer}$ was defined as a mean absolute error ratio described below.

(29) $$I_{maer}=\frac{1}{700}\overset{700}{\underset{i=1}{\sum }}\left|\frac{{\tilde{W}}_{c,d,i}}{W_{c,d,i}}\right|$$

### 2.7. Flow of This Experiment

**Figure 3:**
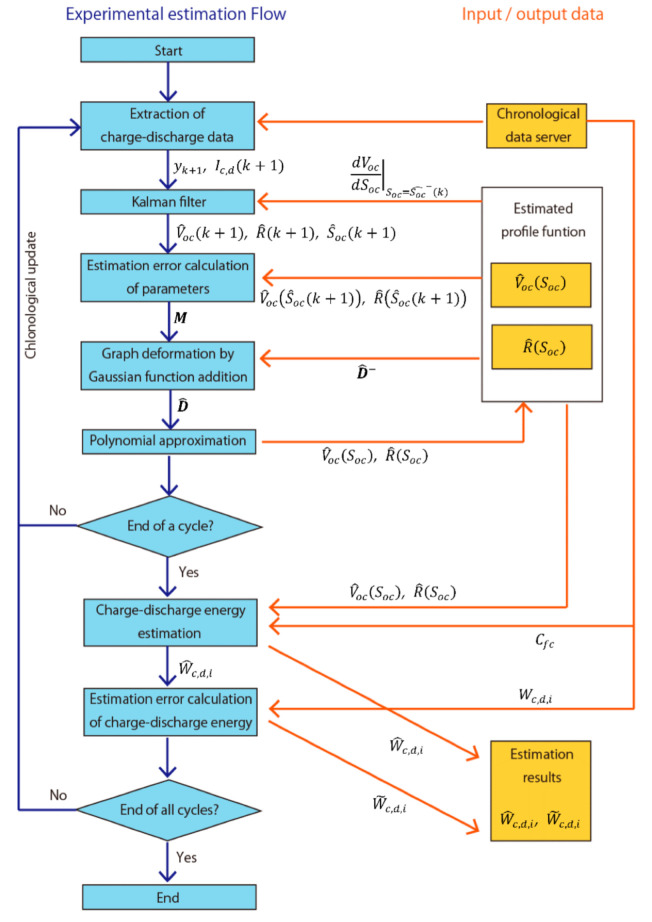
Flow chart of this experiment and data of input and output.

Based on the above description, the flow of this study is described in Figure 3.

${\hat{W}}_{c,d,i}$ and ${\tilde{W}}_{c,d,i}$ were calculated from the charge–discharge chronological data by the series of this estimation process. Then, error index $I_{maer}$ was calculated by Equation (29).

### 2.8. Measuring Instruments and Mathematical Software

**Figure 4:**
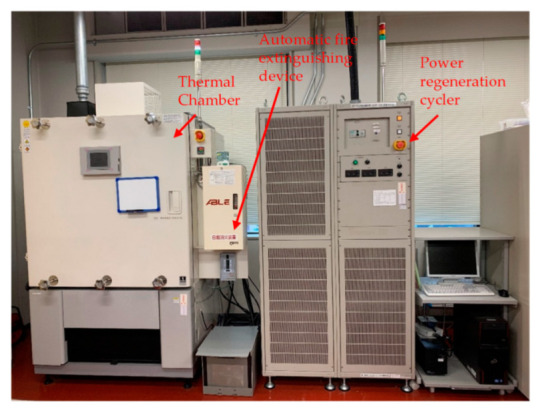
Measuring instruments (power regeneration cycler and thermal chamber).

A charge–discharge cycle test was carried out by using a power-regeneration cycler (Fujitsu Telecom Networks) and a CH43-15P thermal chamber (Nagano Science) as shown in Figure 4. The processes of the Kalman filter, profile-graph deformation, charge–discharge energy estimation, and estimation error and accuracy analysis were carried out using MATLAB 2022a.

## 3. Results

**Figure 5:**
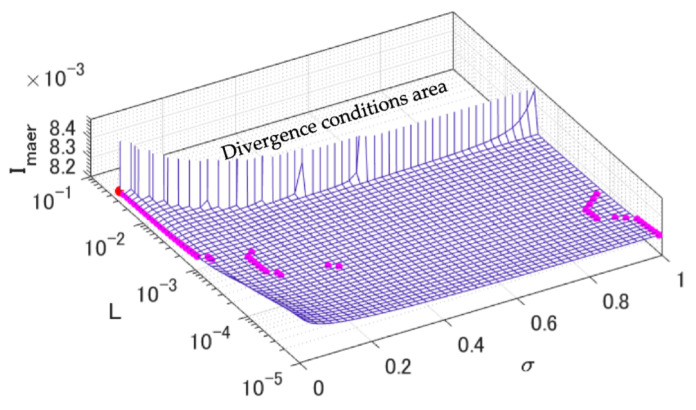
Relationships between the combination conditions of learning rate L and rough range of graph deformation σ, and error indices $I_{maer}$. Magenta-colored dots show best accuracy of each condition of L, and red-colored dot shows the best accuracy of overall conditions.

The combination conditions of learning rate L and rough range of deformation σ are empirically known to be affected by the accuracy of charge–discharge energy estimation [32], so we first analyzed the relationship between these conditions and estimation error indices $I_{maer}$. In this analysis, the actual initial $V_{oc}\left(S_{oc}\right)$ was applied as the reference for $C_{k}$ in the Kalman filter process at the beginning of the 1st cycle. The result of this is shown in Figure 5. The estimation processes were diverged in most cases by about $L>10^{-2}$ and were closer to convergence by reducing σ. This was mainly because larger values of L and σ would have resulted in the accumulation of the excess amount of $\tilde{E}$ that was input to the profile-graph deformation. In addition, the tendency of optimal σ changed with $L=10^{-3.8}$ and $L=10^{-3.2}$ as the boundaries; therefore, the combination conditions of L and σ could be roughly divided into the three groups of “Slow”, “Medium”, and “Fast” as shown in Table 2.

In the next stage, the error of the OCV profile data was artificially adopted and applied as the initial reference for $C_{k}$ in the Kalman filter process. This assumption meant the uncertainty of the initial OCV profiles, which would be affected in the case of reused LIBs. When a reusable LIB starts to be used, the current OCV value and perhaps the electrode materials used in a cell might be known; nevertheless, the overall $V_{oc}\left(S_{oc}\right)$ profile would not. Therefore, ${\hat{V}}_{oc}\left(S_{oc}\right)$ has to be guessed from limited information of the LIB.

**Figure 6:**
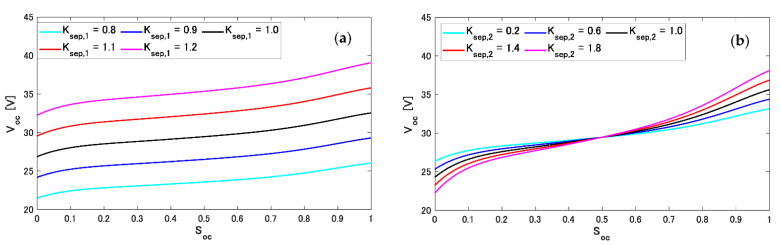
Representations of initial ${\hat{V}}_{oc}\left(S_{oc}\right)$ references separately generated by $K_{sep,1}$ and $K_{sep,2}$. (**a**) The case of Equation (30). (**b**) The case of Equation (31).

To numerically dissociate the initial OCV reference ${\hat{V}}_{oc}\left(S_{oc}\right)$ from the actual initial $V_{oc}\left(S_{oc}\right)$, two methods were used.

(30) $${\hat{V}}_{oc}\left(S_{oc}\right)=K_{sep,1}V_{oc}\left(S_{oc}\right)$$

(31) $${\hat{V}}_{oc}\left(S_{oc}\right)=\left(1+K_{sep,2}\right)V_{oc}\left(S_{oc}\right)-K_{sep,2}V_{oc}\left(0.5\right)$$

where $K_{sep,1}$ and $K_{sep,2}$ are the adjustment coefficients. Equation (30) is the process to increase ($K_{sep,1}>1$) or decrease ($K_{sep,1}<1$) the values of ${\hat{V}}_{oc}\left(S_{oc}\right)$ in one direction. On the other hand, Equation (31) is the process to rotate around $S_{oc}=0.5$ as the central axis. The representations of Equations (30) and (31) are shown in Figure 6. In this figure, the black lines are the actual $V_{oc}\left(S_{oc}\right)$ values, and the other colored lines are deformed and separated from the actual values by $K_{sep,1}$ and $K_{sep,2}$. The equation combining these two separation elements is expressed as shown below.

(32) $${\hat{V}}_{oc}\left(S_{oc}\right)=\left(K_{sep,1}+K_{sep,2}\right)V_{oc}\left(S_{oc}\right)-K_{sep,2}V_{oc}\left(0.5\right)$$

**Figure 7:**
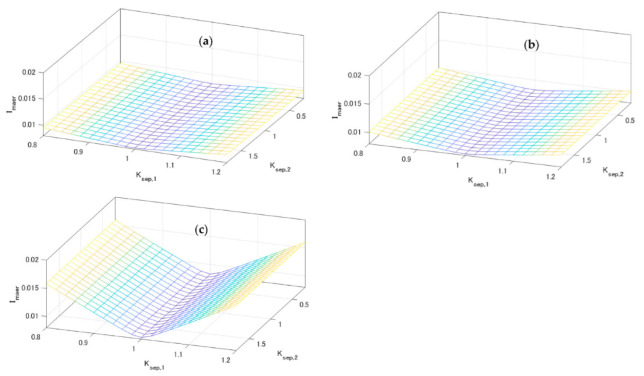
Charge–discharge energy estimation error indices $I_{maer}$ corresponding to various values of $K_{sep,1}$ and $K_{sep,2}$. (**a**) The case of fast condition ($L=10^{-2.3},\;\sigma =0.015$). (**b**) The case of medium condition ($L=10^{-3.45},\;\sigma =0.19$). (**c**) The case of slow condition ($L=10^{-4.4},\;\sigma =0.95$).

Next, the charge–discharge energy estimation during 700 cycles was carried out using various $K_{sep,1}$ and $K_{sep,2}$, and representative L and σ of each condition group in Table 2. This analysis was focused on the adaptability of OCV estimation error ${\tilde{V}}_{oc}\left(S_{oc}\right)$, which was generated with Equation (32). The results of this are shown in Figure 7. From the figure and Equation (32), $K_{sep,1}$ affected the mean absolute error ratio of charge–discharge energy estimation error $I_{maer}$, but $K_{sep,2}$ did not. Greater deviations of $K_{sep,1}$ from 1 increased $I_{maer}$ more. This increasing tendency of $I_{maer}$ became stronger with the decrease in the speed of deformation, that is, when L was smaller.

**Figure 8:**
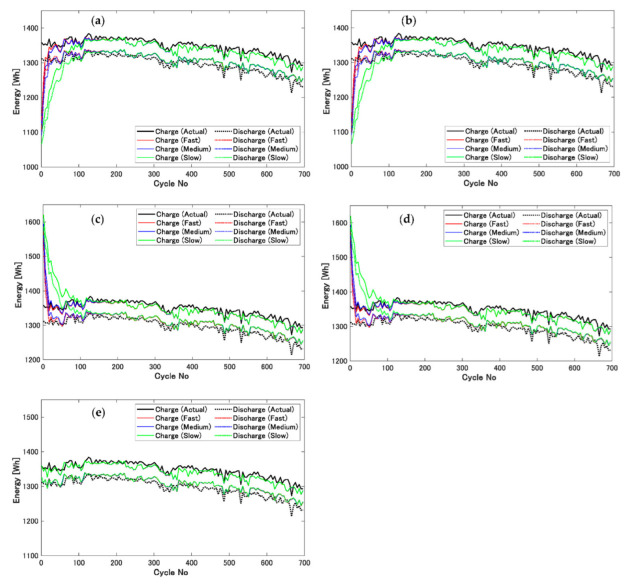
Results of charge–discharge energy estimation starting from incorrect initial OCV profile generated from Equation (32). (**a**) The case of $K_{sep,1}=0.8$ and $K_{sep,2}=0.2$. (**b**) The case of $K_{sep,1}=0.8$ and $K_{sep,2}=1.8$. (**c**) The case of $K_{sep,1}=1.2$ and $K_{sep,2}=0.2$. (**d**) The case of $K_{sep,1}=1.2$ and $K_{sep,2}=1.8$. (**e**) The case of $K_{sep,1}=K_{sep,2}=1$, that is, the estimation was started from the correct initial OCV profile.

On the basis of these observations, we next considered the charge–discharge energy estimation of each cycle. This was carried out starting from the incorrect initial OCV profile, generated by abysmal values of $K_{sep,1}$ and $K_{sep,2}$. These incorrect OCV profiles were generated using Equation (32). In addition, it was carried out with the graph deformation conditions described in Table 2. In this consideration, the estimation starting from the correct initial OCV profile was performed for comparison. The results are shown in Figure 8. The black lines and dotted lines in this figure are the actual transitions of charge–discharge energy of each cycle. The lines and dotted lines in red, blue, and green are the estimated charge–discharge energy values of each cycle calculated as ${\tilde{W}}_{c,d}$ using Equation (25). The transition of charge–discharge energy estimation was affected by $K_{sep,1}$ and not by $K_{sep,2}$, as can be naturally understood from Figure 7. $K_{sep,1}$ was the determinant factor of the sign of the initial charge–discharge energy estimation error. If $K_{sep,1}$ had been lower than 1, the estimated values would have started from lower values than the actual values. In contrast to this, a $K_{sep,1}$ higher than 1 would have resulted in the estimated values starting from higher values. The number of cycles until error convergence was affected by graph deformation speed as described in Table 2. In the cases of fast, medium, and slow deformation speeds, the numbers of cycles required for convergence were almost 20, 40, and 100, respectively. After 100 cycles, the transitions of the estimation of all conditions were homogenous to the ones obtained from the correct OCV profile, as described in Figure 8e. From this, we can see that this proposed method could estimate charge–discharge energy per cycle and calculate the energy efficiency. It should be emphasized that this estimation could be carried out by starting from incorrect and unknown initial profiles of OCV and R assumed for reused LIBs. As discussed above, our purpose was to establish a technique for the high efficiency operation of aggregated reusable LIBs. It can be summarized that this proposed method could realize it.

## 4. Discussion

The Kalman filter is an excellent method for estimating the real-time state of LIBs [27,28,33], although it can only output the current state. This is mainly because it includes recursive processes, which would otherwise be excellent features. In this study, we adopted the graph deformation method by Gaussian function addition [32] to solve this technical problem. This combination was effective to estimate the OCV and R profiles using the Kalman filter. The degradation tendency of LIBs depends on the cell-size formats [35,36]. It reminds us of the difference in learning the followability of the estimation between large and small cells. However, the learning speed of this method is significantly faster than degradation, as shown in Figure 8a–d. Therefore, profiles can be learned in the same way regardless of the cell-size formats.

**Figure 9:**
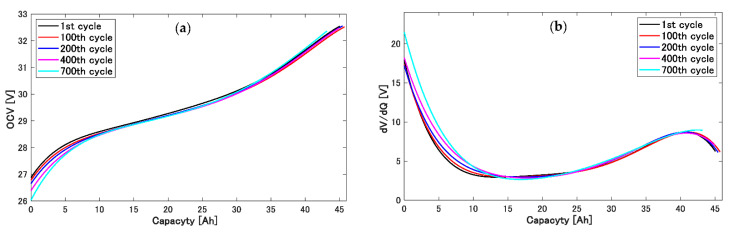
Transition of estimated OCV and dV/dQ profiles. OCV profiles were estimated using the Kalman filter and graph deformation during each cycle. dV/dQ profiles were calculated from the approximate functions of OCV profiles. (**a**) OCV profiles. (**b**) dV/dQ profiles.

We found two beneficial aspects of this method. First, there is the possibility of the Kalman-filter-based differential analysis of the OCV. In general, LIBs are discharged by low currents, and the discharge electric capacity is differentiated with the discharge voltage or inversely differentiated [14,37,38]. In this process, low-current discharge aims to reduce the polarization; in consequence, its chronological discharge data are approximated to the OCV. This differential analysis is known to be effective in diagnosing the internal degradation state of LIBs, for example, the transition of the operation window of positive and negative electrodes [14,37,39]. Although it is necessary that the long discharge time and operation differ from the actual values, this would be difficult to apply to actual aggregated LIBs. Contrary to this, the proposed method could continuously estimate OCV profiles during LIB operation with degradation; therefore, it is suggested that a real-time differential analysis of the OCV can be realized. The transition of the OCV profiles, which was estimated in the case of $K_{sep,1}=K_{sep,2}=1$ and the medium deformation speed described in Table 2, and their differential curves are shown in Figure 9. The OCV profiles of each cycle were approximated as the 6th polynomials shown above in Equation (23); as a result, the subtleties in the curves were not expressed. Simultaneously, differentiation profiles dV/dQ, which were calculated from the estimated OCV functions in Figure 9a, did not have sufficient feature peak information as shown in Figure 9b. It could be the task of a future study to generate differentiated OCV profiles that include sufficient information to diagnose the degradation of LIBs.

Next, information of the OCV function is generally required for the state estimation of LIBs using the Kalman filter. This corresponds to $dV_{oc}∕dS_{oc}$, as previously described in Equations (14) and (15). Naturally, it could be thought that the correct OCV profile is preferable for the accurate estimating and filtering processes. It should be emphasized that the $S_{oc}$ values were also estimated by these processes; in addition, the estimated $S_{oc}$ values were used for the decision of $\;S_{oc,0}$, that is, the center coordinate of graph deformation on the SOC axis. Nevertheless, the wrong OCV profile that was generated by Equation (32) was initially adopted; consequently, it resulted in the accurate convergence of the OCV profiles and the adaptive estimation to the actual charge–discharge energy of each cycle, as previously shown in Figure 8. This result implicitly leads us to the suggestion that the Kalman filter for the state estimation of LIBs has the robustness to the incorrect OCV function references. Again, it could be the object of a future study to validate the adaptability of the proposed method against the clutter of aggregated LIBs, including reused ones.

## 5. Conclusions

We propose a new method that combines the Kalman filter and profile-graph deformation by Gaussian function addition to estimate the charge–discharge energy efficiency per cycle of LIBs. The contributions of this study can be summarized as:

(1) The realization of the characteristic profile estimation of LIBs based on the Kalman filter known as the real-time state estimation method, which can only estimate the scalars of a moment in general;
(2) The successful presentation of the conforming charge–discharge energy estimation that is started from unknown and temporarily decided profiles of the OCV and R assumed as the clutter of aggregated LIBs, including reused ones;
(3) The experimental confirmation of the validity of the recursive references of the estimated SOC, OCV, and R of the combined method of the Kalman filter and profile-graph deformation, which was designed for charge–discharge energy estimation.

In our future work, we aim to apply this method to the efficient operation of aggregated LIBs for the establishment of a system for the maximization of economic gain and renewable energy use.

## Figures and Tables

**Table 1:** The details of cycle test of an 8-series LIB module.

Sample Details	8-Series LIB Module (NiMnCo Cathode and C Anode)
Typical characteristics	29.6 V, 50 Ah, 1.48 kWh
Input and output power	1085 W (constant power)
Range of state of charge	From 0 to 1, based on the manufacturer’s specification
Range of module voltage	From 21.6 V to 33.2 V
Charge–discharge cycles	700 cycles
Measurement time resolution	10 sec
Temperature of cycle test	Room temperature

**Table 2:** Condition groups of profile-graph deformation speeds.

Condition Group	L	σ
Slow	$10^{-5}\leq L\leq 10^{-3.8}$	0.91≤σ≤0.99
Medium	$10^{-3.7}\leq L\leq 10^{-3.2}$	0.11≤σ≤0.27
Fast	$10^{-3.1}\leq L\leq 10^{-1.5}$	0.01≤σ≤0.02

## Data Availability

Not applicable.
